# CheNER: chemical named entity recognizer

**DOI:** 10.1093/bioinformatics/btt639

**Published:** 2013-11-13

**Authors:** Anabel Usié, Rui Alves, Francesc Solsona, Miguel Vázquez, Alfonso Valencia

**Affiliations:** ^1^Department of Basic Medical Science (CMB), University of Lleida & IRBLleida, ^2^Department of Computers an Industrial Engineering (DIEI), University of Lleida, Lleida and ^3^Structural Biology and Biocomputing Programme, Spanish National Cancer Research Center (CNIO), Madrid, Spain

## Abstract

**Motivation:** Chemical named entity recognition is used to automatically identify mentions to chemical compounds in text and is the basis for more elaborate information extraction. However, only a small number of applications are freely available to identify such mentions. Particularly challenging and useful is the identification of International Union of Pure and Applied Chemistry (IUPAC) chemical compounds, which due to the complex morphology of IUPAC names requires more advanced techniques than that of brand names.

**Results:** We present CheNER, a tool for automated identification of systematic IUPAC chemical mentions. We evaluated different systems using an established literature corpus to show that CheNER has a superior performance in identifying IUPAC names specifically, and that it makes better use of computational resources.

**Availability and implementation:** http://metres.udl.cat/index.php/9-download/4-chener, http://chener.bioinfo.cnio.es/

**Contact:** miguel.vazquez@cnio.es

**Supplementary information:** Supplementary data are available at *Bioinformatics* online.

## 1 INTRODUCTION

Automated NER (named entity recognition) of chemical compounds is receiving increased attention from researchers because it can facilitate the application of information extraction to the pharmaceutical treatment of diseases and to understanding how those compounds modulate gene/protein activities. Chemical NER draws from the experience in performing gene and protein NER (Smith, 2008), but differs from it in three ways.

First, catalogs of names and compositions of chemical compounds have been traditionally less accessible. Fortunately, freely available chemical databases such as PubChem (Li *et al.*, 2010) or DrugBank (Wishart *et al.*, 2007) are helping to correct this issue. This makes it possible to do NER of common drug names such as ‘*Aspirin*’ or ‘*Acetone*’ by using a dictionary-based approach.

Second, the complexities and the variability in the morphological structure of systematic IUPAC (International Union of Pure and Applied Chemistry) chemical names (McNaught and Wilkinson, 1997) make it impossible to create a finite dictionary of such names. This poses the main challenge for NER of chemical names (Vazquez *et al.*, 2011). IUPAC names can be simple words, or contain different punctuation marks, sequences of numbers separated by commas and so forth. They can also be combined in different forms (e.g. ‘*18-bromo-12-butyl-11-chloro-4,8-diethyl-5-hydroxy-15-methoxy*’), making it impossible to enumerate them all. This means that NER of such names cannot be done using a dictionary matching, requiring alternative approaches.

Third, systematic nomenclatures of chemicals, like IUPAC, can be used directly to unambiguously derive their chemical structure.

The number of applications that are freely available to do NER of common and systematic names of chemical compounds is still incipient, and their usability, efficiency and accuracy are far from perfect. To help alleviate these problems, in this work we present and benchmark CheNER, a machine learning application based on conditional random fields (CRFs) that performs NER of IUPAC chemical entities with improved performance over comparable tools.

## 2 METHODS

CheNER uses linear CRFs to predict the locations of IUPAC entity mentions in text. CRFs are a probabilistic framework for the labeling or segmentation of sequential data (Lafferty *et al.*, 2001).

The training and benchmarking of the application was done using the corpora provided by Kolářik and Klinger (Klinger *et al.*, 2008; Kolářik *et al.*, 2008). The corpora are divided into a training corpus (***TrainC***, 463 abstracts, 5072 annotated entities), a Medline test corpus with a small number of entities (***MedlineC***, 1000 abstracts, 165 annotated entities) and an evaluation corpus with a large number of entities (***EvalC***, 100 abstracts, 1310 annotated entities). All corpora contain annotated chemical entities written using the IUPAC nomenclature and other types of chemical names. CheNER’s CRF was trained on ***TrainC***. Its performance was subsequently evaluated independently on both, ***MedlineC*** and ***EvalC***.

In training our CRF, we defined a set of features and tested different combinations of them, together with two types of tokenization (A: by spaces, B: by punctuation marks), different orders of CRF (1 or 2) and different sizes of offsets conjunction or sliding windows (0,1), which creates a new additional feature of a token by conjoining its features with those of the *n* (*n* = 0, *n* = 1) surrounding tokens. We then selected the best combination, indicated by the highest *F*-score value obtained in cross-validation over the training set, as a model to use in the evaluation. The selected model performs with an *F*-score value of 80.20% (precision: 82.84%; recall: 77.74%), uses a second order CRF, an offset conjunction of 1, tokenization type A and a particular set of features described in the Supplementary Materials. To mark chemical mentions and establish borders between tokens during training, we used the IOB labeling scheme (Vazquez *et al.*, 2011). Details about the tested sets of features, training and evaluation corpora, training process, modeling assumption, performance and selection are described in Sections 1–3 of the Supplementary Materials.

## 3 RESULTS

### 3.1 Comparative performance for NER of chemical names

The predictive capability of CheNER for IUPAC names was evaluated using the ***EvalC*** and the ***MedlineC*** corpora, performing the evaluation by comparing the system output with a gold standard in terms of the precision (p), recall (r) and *F*-score (F).

**Fig. 1.**
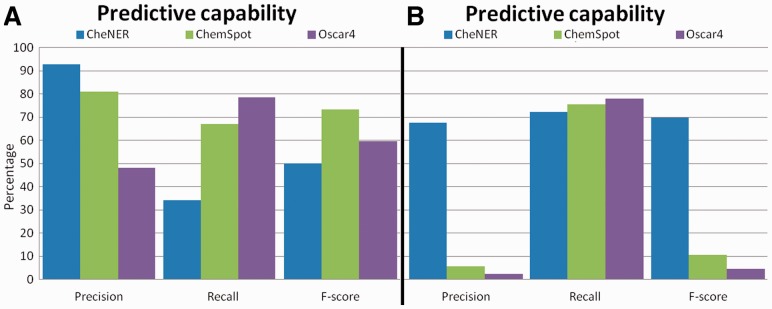
Predictive capability of the different tools identifying IUPAC entities over (**A**) the ***EvalC*** corpus and (**B**) ***MedlineC*** corpus. We measure the ability of the three tools to specifically identify IUPAC chemical entities in the two corpora

There are, to our knowledge, only two other freely available tools for chemical NER. These are ChemSpot (Rocktäschel *et al.*, 2012) and OSCAR4 (Jessop *et al.*, 2011). To compare CheNER’s performance with that of those tools, we use the three applications to independently annotate ***MedlineC*** and ***EvalC*** and compare the results. Our analysis shows that CheNER outperforms the other two applications in the experiments regarding IUPAC names alone (see Fig. 1) due to the fact that it was trained specifically for them. Note that OSCAR4 and ChemSpot do not differentiate between IUPAC and other types of chemical entities and will detect entities that, albeit chemical, will not be IUPAC and will register as false positives. To make the three methods comparable, we ignore non-IUPAC entities that are annotated in the corpora when evaluating performance. Unfortunately the ***MedlineC*** corpus does not annotate non-IUPAC entities, so this corpus can only be compared in terms of recall. We find that CheNER performs better than OSCAR4 and ChemSpot in identifying IUPAC names. Details are given in Section 4 of Supplementary Materials.

Given that CheNER has been trained in the specialized task of recognizing IUPAC names, it is not surprising that when applied to non-IUPAC names it does not perform at the levels of other systems (see Section 4 of Supplementary Materials).

### 3.2 Comparative use of computational resources

We also evaluated how efficiently ChemSpot, OSCAR4 and CheNER use computing resources. We found that CheNER requires less physical memory, running in computers that have <3 GB of RAM, compared with minimum of 3 and 12 GB of RAM required by OSCAR4 and ChemSpot, respectively (see Supplementary Figs S3 and S4 and Section 4 of the Supplementary Materials for details).

## 4 DISCUSSION

Because IUPAC names are the standard in important types of documents, such as patents, and the chemical structure is often derivable from the mention itself, it is important to have an application specifically devised for their identification. Given the potentially infinite number of IUPAC entities, it is not feasible to develop a dictionary-based approach to identify them, and natural language processing methods are more suitable to identify those entities. Thus, we developed CheNER, an NER approach for finding IUPAC names in text, using CRFs. We demonstrate that CheNER annotates IUPAC names in documents with a better *F*-score than ChemSpot and OSCAR4. CheNER is the only tool that is specifically developed to identify only such names, whereas ChemSpot and OSCAR4 do not differentiate between entity types.

We also show that CheNER needs less memory and CPU than the others to perform the same tasks. In addition, CheNER is self-contained, requiring only that Java is installed to run, which makes it easier to integrate in other systems.

## Supplementary Material

Supplementary Data

## References

[btt639-B2] Jessop D (2011). OSCAR4: a flexible architecture for chemical text-mining. J. Cheminform..

[btt639-B3] Klinger R (2008). Detection of IUPAC and IUPAC-like chemical names. Bioinformatics.

[btt639-B4] Kolářik C (2008). Chemical names: terminological resources and corpora annotation. Proceedings of Workshop on Building and Evaluating Resources for Biomedical Text Mining.

[btt639-B5] Lafferty J (2001). Conditional random fields: probabilistic models for segmenting and labeling sequence data. Proceedings of the Eighteenth International Conference on Machine Learning (ICML 2001).

[btt639-B6] Li Q (2010). PubChem as a public resource for drug discovery. Drug Discov. Today.

[btt639-B7] McNaught AD, Wilkinson A (1997). IUPAC Compendium of Chemical Terminology. Gold Book.

[btt639-B8] Rocktäschel T (2012). ChemSpot: a hybrid system for chemical named entity recognition. Bioinformatics.

[btt639-B9] Smith L (2008). Overview of BioCreative II gene mention recognition. Genome Biol..

[btt639-B10] Vazquez M (2011). Text mining for drugs and chemical compounds: methods, tools and applications. Mol. Inform..

[btt639-B11] Wishart DS (2007). DrugBank: a knowledgebase for drugs, drug actions and drug targets. Nucleic Acids Res..

