# Supplementary material for: CheNER: chemical named entity recognizer
Source: Bioinformatics. 2013 Nov 13;30(7):1039–40. doi: 10.1093/bioinformatics/btt639 (PMC3967102; doi:10.1093/bioinformatics/btt639)
Supplement: Supplementary Data [file supp_30_7_1039__index.html]

CheNER: Chemical Named Entity Recognizer — CheNER: chemical named entity recognizer — CheNER: chemical named entity recognizer — Supplementary Data 

# CheNER: chemical named entity recognizer

## Supplementary Data

files

**Files in this Data Supplement:**

- Supplementary Data - docx file
