# Supplementary material for: CheNER: chemical named entity recognizer
Source: Bioinformatics. 2013 Nov 13;30(7):1039–40. doi: 10.1093/bioinformatics/btt639 (PMC3967102; doi:10.1093/bioinformatics/btt639)
Supplement: Supplementary Data [file supp_btt639_Supplementary_Material_28102013.docx]

| Data and Text-Mining  Supplementary Document of CheNER: Chemical Named Entity Recognizer  Anabel Usié^1,2^, Rui Alves^1,*^, Francesc Solsona^2^, Miguel Vázquez^3,*^ and Alfonso Valéncia^3,*^  ^1^Department of Basic Medical Science (CMB), University of Lleida & IRBLleida, Lleida Spain.  ^2^Department of Computers and Industrial Engineering (DIEI), University of Lleida, Spain.  ^3^Structural Biology and Biocomputing Programme, Spanish National Cancer Research Center (CNIO), Madrid, Spain. |
| --- |

**Section S1 to S4.**

**Figures. S1 to S4**

**Section 1. Features set used**

The set of features used to train the CRF model is shown in Supplementary Table 1. Some of these features were identified by previous studies as being the most discriminative in the identification of gene and protein names (Klinger *et al.*, 2008).

**Supplementary Table 1.**Types of features used

| Feature | Description |
| --- | --- |
| Morphological Features*(MF)* | Identifies specific features of words. For example: is the word all caps? does it contain a number? dashes, slashes or other punctuation marks? |
| Prefixes/Suffixes *(PS)* | Identifies specific prefixes or suffixes of a given length (2, 3, 4 characters) that are common in chemical names. |
| Types of Characters*(ToC)* | Identifies specific types of characters that are more common in chemical names, such as Greek letters, roman numbers, etc. |
| Length *(L)* | Classifies tokens by length. If the length is less than 5, the token is Short. If length is between 5 and 15, the token is Medium, otherwise, the token is Large. |
| Word class *(W)* | Analyzes the structure of chemical names in terms of frequency of upper and lower case characters, digits and other types of characters. |
| Brief Word class *(WB)* | Same as W, collapsing consecutive identical types of character into one Examples of these two features: *i.e. 1-methyl: 0.aaaaaa and.1-methyl: 0.a*. |
| List *(BNS/SW)* | Matches token to basic name segments and to the stop words list. |

**Section 2. Detailed information about the corpora used.**

These corpora include chemical entities of different types: IUPAC and PARTIUPAC employ multi-word systematic names and partial chemical names (A D McNaught and Wilkinson, 1997), MODIFIER names classify chemical modifiers (NCI Thesaurus), FAMILY type includes generic chemical entities such as “purine”, or “sugar”, (Klinger *et al.*, 2008), ABB type includes those chemicals that are abbreviations of names (Abbr. and Symb., 1967),SUM type includes the chemical and/or atomic formulas for the different compounds (A D McNaught and Wilkinson, 1997), and TRIVIAL are common names composed by single word terms. Supplementary Table 2 summarizes the entity composition of the three corpora.

**Supplementary Table 2.**Chemical entity types

| Chemical types | ***TrainC*** | ***EvalC*** | ***MedlineC*** |
| --- | --- | --- | --- |
| IUPAC & PARTIUPAC | 4033 | 483 | 151 |
| MODIFIER | 1039 | 104 | 14 |
| FAMILY | 0 | 99 | 0 |
| ABB | 0 | 161 | 0 |
| SUM | 0 | 49 | 0 |
| TRIVIAL | 0 | 414 | 0 |
| **Total** | **5072** | **1310** | **165** |

While being an invaluable resource for this kind of work, and the best dataset to date for this task, close examination of these corpora reveals a caveat: while ***TrainC*** and ***MedlineC*** only have 3 entities in common, 46.16% of the entities in ***EvalC*** are also found in ***TrainC***. To our knowledge this fact had not been highlighted before.


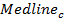

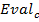

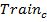


**Section 3. The training process**

First, we look for the combination of parameters and features sets (modeling assumptions) that create the model with the best F-Score performance. The performance of each modeling assumption was assessed using 5-fold cross-validation over the ***TrainC*** corpus. In cross validation, the dataset is randomly divided into several equally sized chunks or folds (5 in our case), and each fold is in turn used to validate a model trained using the other folds. Second, once we have determined the best model assumption as the one with the highest averaged F-Score performance over all 5 folds, it is used for the final training over the complete ***TrainC*** corpus.


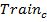

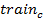


The different modeling assumptions are detailed in Table 3. Each modeling assumption explores combinations of 3 different characteristics. First, two tokenization types were used. In type A only blank spaces were used to delimit tokens, while in type B several other characters were also considered as possible token delimiters (see Supplementary Table 3 for details). Second, CRFs of order 1 and 2 were used for the training. Third, two values of OC were considered (0: no context considered, and 1: features from tokens that immediately precede and follow the token of interest are considered in predicting that token’s label). We obtained a top F-Score performance of 80.20% (Precision: 82.84%; Recall: 77.74%), using a 2nd order of CRF, an offset conjunction of 1, tokenization type A (by spaces), and the following set of features: MF, Toc, PS, W, WB, L, BNS and SW.

**Supplementary Table 3.** Modeling assumptions for the CRF model.

| Training configurations | Tokenization type | Order CRF | OC |
| --- | --- | --- | --- |
| 1 | A | 1 | 0 |
| 2 | A | 1 | 1 |
| 3 | A | 2 | 0 |
| 4 | A | 2 | 1 |
| 5 | B | 1 | 0 |
| 6 | B | 1 | 1 |
| 7 | B | 2 | 0 |
| 8 | B | 2 | 1 |

For each modeling assumption all the features set were used (see Table 1). A: Uses blank spaces as delimiters of tokens. B: Uses blank space, punctuation marks (dots, dashes, etc) and parenthesis as delimiters if tokens.

**Section 4. Comparison of CheNER to other chemical tools.**

A set of four experiments was conducted to benchmark the performance of different tools performing chemical NER: (1) IUPAC entities in ***EvalC***, (2) IUPAC entities in ***MedlineC***, (3) all entity types in ***EvalC***, and (4) all entity types in ***MedlineC***. The tools that were comparatively evaluated with respect to CheNER were OSCAR4 (Corbett and Murray-Rust, 2006; Jessop *et al.*, 2011) and ChemSpot (Rocktäschel *et al.*, 2012).

The results obtained in experiment 1 and 2, the identification of IUPAC names, are shown in Figure 1A and 1B, respectively (see main document), and in Supplementary Table 4. CheNER has the best global performance in experiments 1 and 2, as measured by the F-score. To make the comparisons reliable, we eliminate all non-IUPAC entities annotated in each corpora by OSCAR and ChemSpot. This must be done to account for the fact that ChemSpot and OSCAR4 do not differentiate between the types of chemical entities they annotate, which would lead to an artificially low precision for these two applications. In addition we manually check the FP results of each tool. We find that most of these FP are real chemical entities that failed to be originally annotated in the corpora. When these are eliminated and only real FP entities are considered, both ChemSpot and OSCAR4 always reported a higher number of FP entities than CheNER. Thus, CheNER has more precision than either OSCAR4 or ChemSpot, and a comparable recall to either of the other tools. Overall, CheNER has the highest F-score in identifying IUPAC chemical entities.

The results obtained in experiment 3 and 4, are shown in Figures S1 and S2, respectively. As expected, CheNER has the worst F-score performance in experiment 3, as it mostly recognizes IUPAC names.


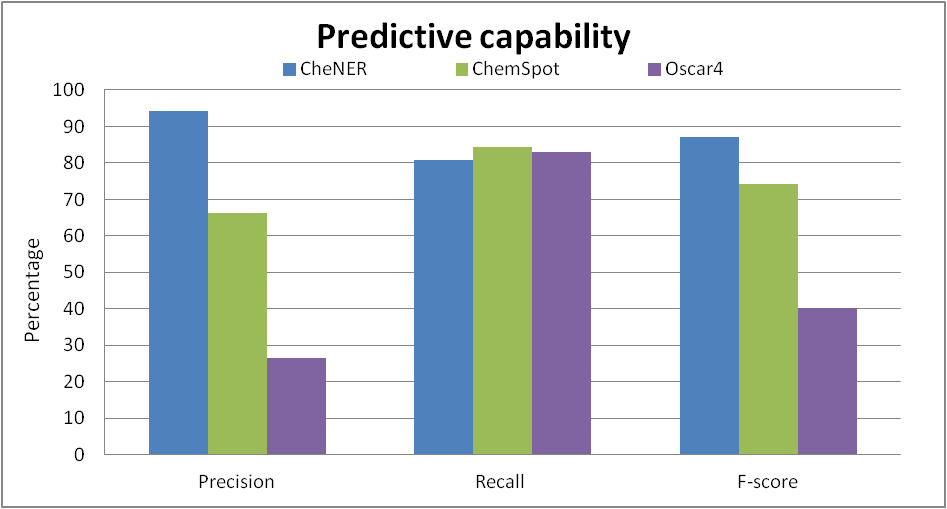


**Figure S1**. Predictive capability of the different tools identifying all type of entities over the EvalC corpus in terms of precision, recall and F-score. In this experiment we measured the F-score of the three tools in identifying all types of chemical entities.

**Figure S2**. Predictive capability of the different tools identifying all type of entities over the MedlineC corpus in terms of precision, recall and F-score. In this experiment we measured the F-score of the three tools in identifying all types of chemical entities.


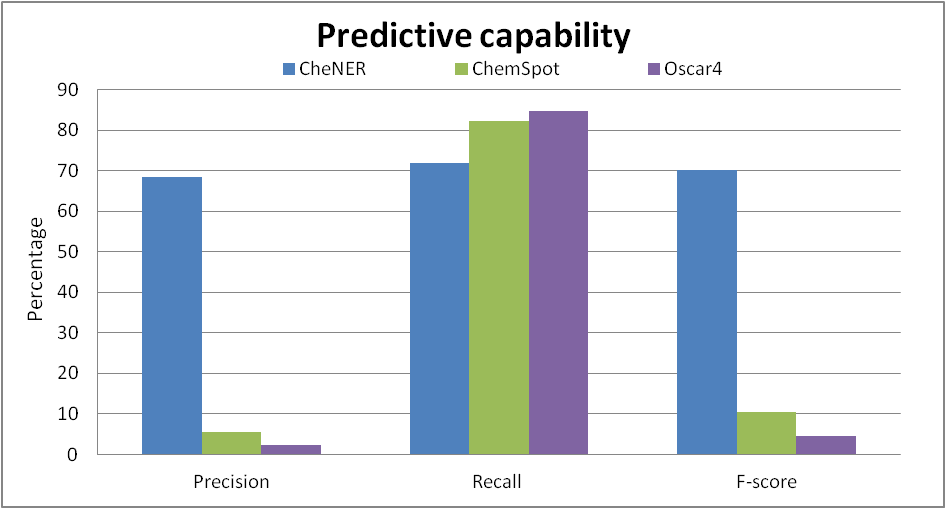


**Supplementary Table 4.** Summary of F-score values for each tool in each experiment.

|  | Exp. 1 | Exp. 2 | Exp. 3 | Exp. 4 |
| --- | --- | --- | --- | --- |
| CheNER | 86.98 | 70.06 | 50.03 | 69.94 |
| Oscar4 | 40.24 | 4.53 | 59.68 | 4.52 |
| ChemSpot | 73.35 | 10.51 | 74.24 | 10.57 |

Exp1: identify IUPAC entities in EvalC; Exp2: identify IUPAC entities in MedlineC; Exp.3 identify all type of entities in EvalC (complete corpus); Exp.4: identify all type of entities in MedlineC (complete corpus).

The low performance of ChemSpot and Oscar4 in MedlineC is a consequence of the way that this corpus is annotated. While only IUPAC and MODIFIER chemical names are annotated, other types of chemicals are also present. A partial manual analysis of the results reveals that many of the false positive entities identified by OSCAR4 and ChemSpot are non IUPAC chemical entities. Therefore, the only real comparison of predictive capability that we can do between the three tools while tagging entities of ***MedlineC*** in this experiment is in term of recall. We show that CheNER has the worst recall in this experiment, mostly due to a failure in identifying MODIFIER entities.

Summarizing the results, CheNER is the application that more accurately identifies IUPAC chemicals names. It is also the only available tool that was specifically developed to identify this nomenclature. In this context, the three tools have a similar recall and CheNER’s outperforms the other tools based on higher precision.

## We also evaluated how efficiently ChemSpot, Oscar4 and CheNER use available computing resources. To do so, we ran each application on the same machine (i7 processor, with four CPUs and 20GB of RAM) and monitored the consumption of main memory and CPU of the system during the annotation of the two evaluation corpora. Suppl. Figure 3 shows that CheNER uses less memory than the others (<1GB-3.5% of available RAM). In contrast ChemSpot uses the most amount of memory (>7GB-35.7%) and OSCAR4 needed an intermediate amount of memory (<2GB-5.5%). To estimate total memory requirements we must consider also the memory required to run the JVM (Java Virtual Machine). Suppl. Figure 2 shows that CheNER also requires less CPU than the other applications. In addition, CPU usage is more constant over time than that of ChemSpot and Oscar.


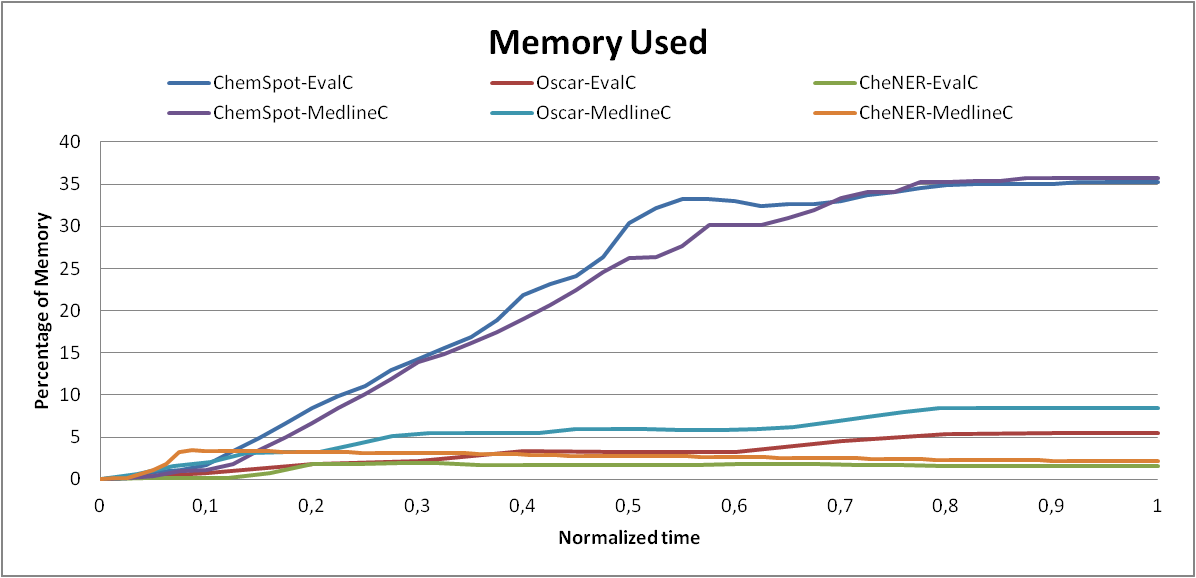


F**igure S3.** Memory consumption for each application during the annotation of both corpora, EvalC and MedlineC. Time was normalized in each run. Absolute time for the same run is similar for the three programs


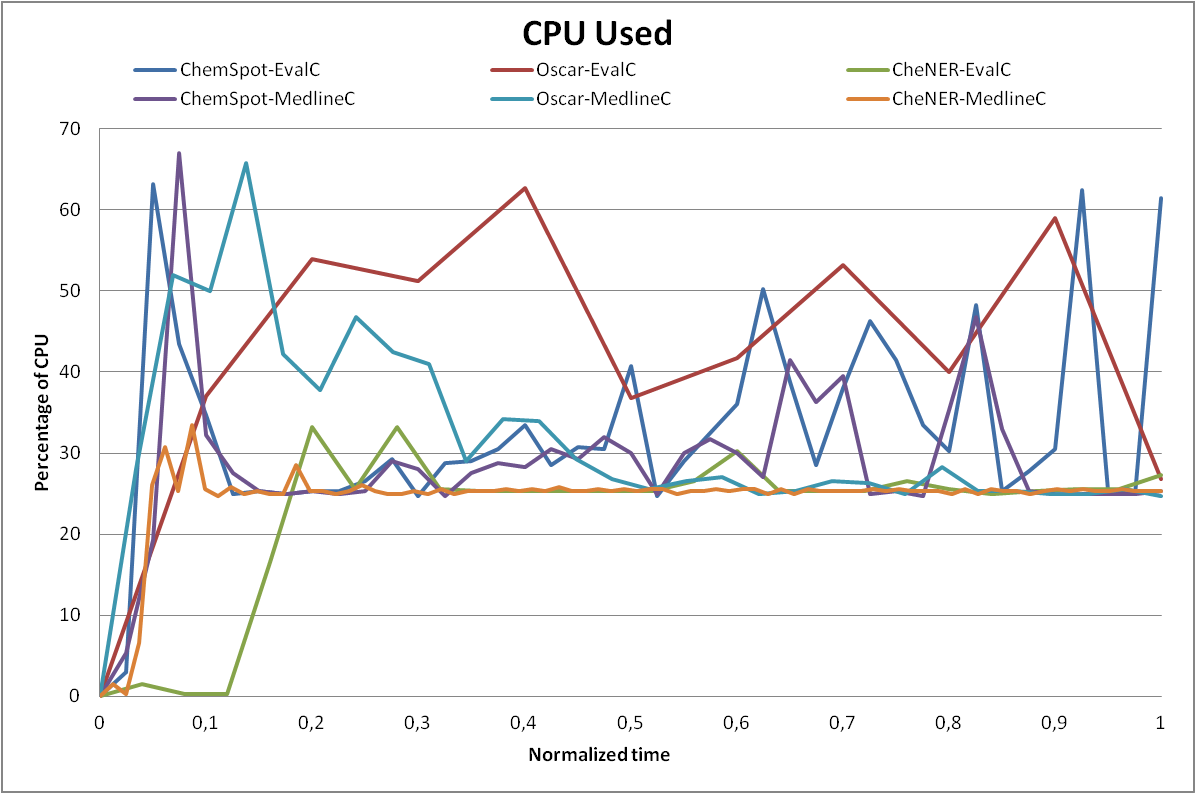


F**igure S4.** CPU consumption for each application during the annotation of the EvalC and MedlineC corpora. Time was normalized in each run. Absolute time for the same run is similar for the three programs.
